# Effect of drug-loaded microbubbles combined with ultrasound on the apoptosis of cancer cells and the expression of Bax and Bcl-2 in a rabbit VX2 liver tumor model

**DOI:** 10.1042/BSR20181144

**Published:** 2019-05-24

**Authors:** Kun Chen, Liang Zhang

**Affiliations:** 1 Department of Ultrasonography, Huashan Hospital Affiliated to Fudan University, Shanghai 200040, P.R. China; 2 Department of Physician, Shuguang Hospital Affiliated to Shanghai University of Traditional Chinese Medicine, Shanghai 200040, P.R. China

**Keywords:** apoptosis, Bax, Bcl-2, drug-loaded microbubbles, ultrasound targeted microbubble destruction

## Abstract

The aim of the present study was to investigate whether the use of drug-loaded microbubbles combined with ultrasound promotes the apoptosis of cancer cells by regulating B-cell lymphoma-2 (Bcl-2) and Bcl-2-associated X protein (Bax) expression. Adriamycin-loaded PLGA nanoparticles (ADM-NP) were fabricated using a modified emulsification process. Lipid microbubbles (NH_2_-MB) were prepared by mechanical vibration. The carboxyl groups of ADM-NP and NH_2_-MB underwent a condensation reaction after 48 h, and adriamycin-loaded PLGA nanoparticles microbubble complexes (ADM-NMC) were obtained. High-performance liquid chromatography demonstrated that the entrapment efficiency and drug loading of ADM-NMC were 85.32 ± 5.41% and 7.91 ± 0.27%, respectively. The VX2 liver cancer model was established in 30 New Zealand rabbits, which were subsequently divided into three groups (*n*=10): a control group that received 5 ml of saline, an ADM-NP group that received 5 ml of ADM-NP and an ADM-NMC group that received 5 ml of ADM-NMC. Rabbits in the ADM-NP and ADM-NMC groups underwent irradiation 120 s with low frequency ultrasound (1 MHz, 0.5 W/cm^2^) for 120 s following injection. The echogenicity of tumors markedly increased following ADM-NP and ADM-NMC treatment. Staining with hematoxylin and eosin demonstrated that the tumor shape became more normal in the ADM-NP and ADM-NMC groups compared with the control group. Immunohistochemical staining and Western blotting determined that the expression of Bax increased and the expression of Bcl-2 decreased following treatment with ADM-NP and ADM-NMC. Cancer cell apoptosis was detected by flow cytometry and it was determined that apoptosis significantly increased following treatment with ADM-NP and ADM-NMC (*P*<0.01). Therefore, the present study demonstrated that the use of drug-loaded microbubbles combined with ultrasound may enhance the efficiency of tumor inhibition. This may be due to the promotion of cancer cell apoptosis via regulation of Bax and Bcl-2 expression.

## Introduction

Hepatocellular carcinoma (HCC) is one of the most common malignancies in the world [1]. A report in 2011 documented that the annual incidence ranges from 50 to 150 cases per 100,000 persons in parts of Africa and Asia, where HCC is responsible for a large proportion of cancer-related deaths [2]. Surgery, radiotherapy and chemotherapy are the current therapeutic strategies used to treat HCC and are often combined to improve patient outcomes [3]. Chemotherapy remains the most common method of treating most types of advanced cancer. However, high interstitial fluid pressure has been an important factor that impairs the efficiency of chemotherapy to tumors [4]. And another obstacle to cancer therapy is the limited distribution of low molecular weight anticancer drugs within the tumor tissues [5]. Additionally, chemotherapeutic drugs must cross the cell membrane and enter the cell to become active. However, this presents a challenge and requires active uptake through plasma membrane transporters, which may not always be present in the target cells [6]. Adriamycin (ADM) is one important type of active chemotherapeutic agent widely used to target malignant tumors. To improve the efficiency of anticancer chemotherapeutics, the technique of microbubble-assisted ultrasound has been developed [7]. Ultrasonic microbubbles may not only help with imaging, but also be used as gene or drug carriers, as they are relatively stable, can carry a high drug load and exhibit high entrapment efficiency [8]. Polylactic-co-glycolic acid (PLGA) is an ideal drug carrier and ADM may be wrapped inside PLGA to form an ADM nanoparticle (ADM-NP). It has been demonstrated that apoptosis in human breast cancer cells may be induced by the use of microbubble contrast agents combined with ultrasound [9]. Furthermore, ultrasound-targeted microbubble destruction, which is a novel, targeted drug delivery technology using ultrasonic microbubbles as a carrier, may increase the effectiveness of drug treatments in targeted regions [10].

Apoptosis is a process of programmed cell death controlled by polygenes and chemotherapeutic drugs induce the apoptosis of tumor cells in chemotherapy to treat cancer [11]. The Bcl-2 gene family, which may be classified into anti-apoptotic proteins, such as B-cell lymphoma-2 (Bcl-2) and pro-apoptotic proteins, such as Bcl-2-associated X protein (Bax), is associated with the apoptotic process [12]. Bcl-2 and Bax produce opposing effects in the regulation of cell apoptosis [13], and the proportion of Bcl-2 and Bax protein determines whether cells survive or undergo apoptosis, which in turn influences the therapeutic effects of different cancer treatments [14].

The present study used adriamycin-loaded PLGA nanoparticles microbubble complexes (ADM-NMC) combined with ultrasound targeted microbubbles destruction techniques to assess the apoptosis of tumor cells in the targeted treatment of VX2 liver tumors in a rabbit model.

## Materials and methods

### Preparation of ADM-NMC

Dipalmitoyl phosphatidylcholine (DPPC) was mixed with amino-polyethylene glycol (peg)-2 stearyl phosphatidylethanolamine (NH_2_-PEG-DSPE; Avanti Polar Lipids Inc., Alabaster, AL, U.S.A.) in a ratio of ∼5:2. The mixture was dissolved in a 1.5 ml of vial containing 50 μl of 100% glycerin and 450 μl of phosphate buffered-saline (PBS; Shanghai Ziyi Reagent Factory, Shanghai, China). The vial was incubated in 45°C water for 30 min. Subsequently, the container was degassed and perfused with perfluoropropane gas (C_3_F_8_, MW 188 g/mol, the department of physical and chemical engineering at Tianjin University, Tianjin, China). The mixture was then mechanically vibrated for 30 s in a dental amalgamator (Shanghai Medical Instruments (Group), Ltd., Corp, Shanghai, China) at a vibration frequency of 60 Hz. Lipid microbubbles (NH_2_-MB) were then harvested and preserved at 4°C.

The 2 mg of ADM (Wuhan Boster Biological Technology, Ltd., Wuhan, China) was dissolved in 1 ml of double-distilled water and this solution was added to dichloromethane solution containing PLGA (molecular weight, 12,000; copolymer ratio 50:50; Jinan Daigang Biomaterial Co., Ltd., Shandong, China). The mixture was sonicated by externally applied 20-kHz ultrasound for 50 s to produce microemulsions. Subsequently, 20 ml of 4% polyvinyl alcohol solution was added to microemulsions using a homogenizer at 1000 r for 5 min. Then, 20 ml of 2% isopropyl alcohol was added to the mixture at room temperature under magnetic stirring for 2 to 5 h. It was rinsed using double-distilled water and 2-(N-Morpholino) ethanesulfonic acid hydrate (MES, pH 6.0) buffer prior to centrifugation at 5000 × ***g*** at 4°C, for 5 min for three times. The final ADM concentration in the PLGA was measured by high-performance liquid chromatography (HPLC). The 10 μl supernatant of ADM-NP was injected into HPLC system. HPLC was performed using an Agilent 1100 (Agilent, Technologies, Inc., Santa Clara, CA, U.S.A.) with a Thermo Synronis C_18_ column (4.6 mm × 150 mm, 5 μM; Thermo Fisher Scientific, Inc., Waltham, MA, U.S.A.). A mobile phase of glacial acetic acid (pH 3.5) and methanol mixed at a volume ratio of 3:7 was used. The flow rate was set at 1.0 ml/min and the analysis wavelength was at 254 nm.

The ADM-NP was dispersed and dissolved in 2 ml of MES buffer (pH 6.0) and was added to 1-(3-dimethylaminopropyl)-3-ethylcarbodiimide hydrochloride/N-Hydroxysuccinimide (EDC/NHS), it was then incubated for 1 h at 4°C and washed three times with MES buffer (pH 8.0). The mixture was centrifuged under the same centrifugation conditions (5000 × ***g*** at 4°C, for 5 min) to remove unreacted EDC/NHS and dispersed and then dissolved in MES buffer (pH 8.0). ADM-NP was added to NH_2_-MB by dripping slowly, was incubated at 4°C for 2 h and the mixture was subsequently kept at 4°C in a refrigerator for 48 h to continue the reaction. The final ADM-NMC concentration (10 μl of ADM-NMC was loaded into the HPLC system) was measured by HPLC as described below. The 10 μl supernatant of ADM-NMC was injected into HPLC system. HPLC was performed using an Agilent 1100 (Agilent, Technologies, Inc., Santa Clara, CA, U.S.A.) with a Thermo Synronis C_18_ column (4.6 mm × 150 mm, 5 μM; Thermo Fisher Scientific, Inc., Waltham, MA, U.S.A.). A mobile phase of glacial acetic acid (pH 3.5) and methanol mixed at a volume ratio of 3:7 was used. The flow rate was set at 1.0 ml/min and the analysis wavelength was at 254 nm.

### Measurement of the drug-loading coefficient and entrapment efficiency of PLGA

The PLGA loading rate and encapsulation efficiency were investigated using an Agilent 1100 HPLC system (Agilent, Technologies, Inc., Santa Clara, CA, U.S.A.) with a Thermo Synronis C_18_ column (4.6 mm × 150 mm, 5 μM; Thermo Fisher Scientific, Inc., Waltham, MA, U.S.A.). A mobile phase of glacial acetic acid (pH 3.5) and methanol mixed at a volume ratio of 3:7 was used. The flow rate was set at 1.0 ml/min and the analysis wavelength was at 254 nm. The standard calibration curve derived from the test exhibited high linearity, accuracy and reproducibility, with a correlation coefficient of 0.9995. The 50 μl supernatant of ADM-NP was injected into HPLC following centrifugation (5000 × ***g*** at 4°C, for 5 min). The drug loading and entrapment efficiency were calculated using the following equations:

Drug-loading rate = free ADM /total ADM × 100%

Drug entrapment efficiency = (total ADM − free ADM)/total ADM × 100%

### Preparation of VX2 tissue fragments

The VX2 tumor-bearing rabbit (*n*=1; female; 15–16 weeks; 2.2 ± 0.2 kg) was purchased from Chongqing Medical University (Sichuan, China). The animals were housed at 20°C, a 12 h light/dark cycle, 60–70% humidity and had free access to food and water. The killing of rabbit was intravenously injected with sodium pentobarbital (Sigma, U.S.A.) (30–40 mg/kg body weight) when the external tumor grew to a size of ∼4 cm in diameter. The tumor was harvested and placed in 0.9% sodium chloride immediately following killing and was stripped to obtain flesh hoary fish-meat like tissue. Subsequently, the necrotic tissues surrounding connective tissue and fat were removed. To obtain tissue fragments, chunks of the excised tumor were cut into 1.0 mm^3^ fragments using ophthalmological scissors. All procedures were performed under sterile conditions.

### Animals and VX2 liver tumor implantation

A total of 30 New Zealand White rabbits (mean weight, 2.1 ± 0.1 kg; 14–15 weeks; females) were purchased from the Animal Experimental Center of Chongqing Medical University. The animals were housed at 20°C, a 12 h light/dark cycle, 60–70% humidity and had free access to food and water. Prior to tumor implantation, animals were sedated with an intramuscularly injection of 35 mg/kg ketamine hydrochloride (Sigma), 5 mg/kg xylazine chloride (Sigma, U.S.A.) and 0.75 mg/kg acepromazine (Sigma). The left lobe of the liver was exposed through a midline abdominal incision and minced pieces of harvested VX2 carcinoma tissue (∼1.0 mm^3^) were locally implanted. Penicillin was administered to rabbits by intramuscular injection for 3 days after the operation (10000 U/kg body weight per day). Tumors were incubated after 2 weeks following tumor implantation. The present study was approved by the Animal Care and Use Committee of Chongqing Medical University.

### Ultrasound imaging

A total of 2 weeks following tumor implantation, experiments were performed on six tumor-bearing rabbits anesthetized by intravenous injection of sodium pentobarbital (Sigma; 30–40 mg/kg body weight) that were randomly divided into three groups (10 animals/group): a control group, an ADM-NP group and an ADM-NMC group. Imaging experiments on tumor-bearing rabbits were performed *in vivo* using a 10-MHz linear transducer (Color Doppler ultrasonography, LOGIQ9; GE Healthcare, Chicago, IL, U.S.A.). Liver scans were used to select optimum sections of tumors according to echo information of the tumor obtained using the fundamental wave mode. Subsequently, ADM-NP and ADM-NMC diluted with physiological saline were injected into rabbits in the ADM-NP and ADM-NMC groups. The control group received physiological saline treatment alone. The ultrasonic features of the tumors in each group were observed in a harmonic way and echo strength was compared among the three groups.

### Grouping and treatments

A total of 30 New Zealand White rabbits with hepatic metastasis of VX2 carcinoma were randomly divided into three groups (*n*=10): a control group, an ADM-NP group and an ADM-NMC group. All groups were treated on days 21, 23, 25, 27 and 29. Rabbits in the control group received an injection of 5 ml of saline via the ear vein; 5 ml of ADM-NP was injected into ADM-NP rabbits in the same way and 5 ml of ADM-NMC was injected via the ear vein in the ADM-NMC group. Rabbits in the ADM and ADM-NMC groups received 120 s irradiation with low frequency ultrasound (UGT1025, Institute of ultrasonic imaging, chongqing medical university; 1 MHz, 0.5 W/cm^2^) following injection. Rabbits were killed by injection of air into the vein on the edge of the ear after the final treatment; the tumor tissue was stripped and subsequently measured.

### Flow cytometry

The tumor tissue from rabbits in the control, ADM-NP and ADM-NMC groups were minced into 5 mm^3^ pieces at the central and edge of the tumor and were used to prepare the monoplast suspension. Flow cytometry was then used to determine tumor cell apoptosis. The FITC Annexin V/PI kit (Thermo Fisher Scientific, Inc.) was used, following the manufacturer’s instructions. A flow cytometry analyzer FACSCalibur with Cell Quest software version 3.1 (BD Biosciences, Franklin Lakes, NJ, U.S.A.) was used to detect cellular apoptosis.

### Hematoxylin and eosin staining

The tumors were fixed in 4% paraformaldehyde for 4 h at room temperature and embedded in paraffin wax immediately following resection. Tissues were routinely processed for paraffin embedding and sections 3 µm thick were cut and placed on glass slides. Tissue samples were stained with hematoxylin and eosin and observed under an inverted microscope. Two experienced pathologists determined the histological type.

### Immunohistochemistry

The paraffin sections of the tumor tissues were conventionally dewaxed by xylene. Endogenous peroxidase was blocked using hydrogen peroxide (3% H_2_O_2_). The slides were blocked with 10% normal goat serum (Beyotime) for 10 min at 37°C. The slides were incubated with primary anti-Bax (1:100, ab216494, Abcam) and anti-Bcl2 (1:100, ab692, Abcam) antibodies at 4°C overnight. Then, the slides were incubated with biotin-labeled secondary antibody at 37°C for 30 min. The immunohistochemical staining was undergone using the avidin-peroxidase complex technique (ABC Kit-vector laboratories, Bur linguine, CA). The ABC reaction was developed in the presence of Diamino Benzedine supplement with hydrogen peroxide (DAB). The slides were observed under an inverted microscope.

### Detection of apoptotic protein expression using Western blotting

Tumor tissue homogenates were lysed using NP40 radioimmunoprecipitation assay buffer (Beyotime, China) plus protease inhibitors (Roche). The homogenate was centrifuged at 700  × ***g*** for 10 min at 4°C and the protein concentration was detected using the bicinchoninic acid protein assay. Proteins (25 μg/lane) were run on 10% sodium dodecyl sulfate (SDS) polyacrylamide gels and then transferred to polyvinylidene difluoride (PVDF) membranes. Nonspecific binding was blocked with 5% nonfat milk (diluted in PBS) for 1 h at room temperature and incubated overnight at 4°C with primary antibodies against GAPDH (1:1,000, ab8245, Abcam), Bcl-2 (1:500, ab692, Abcam) and Bax (1:500, ab216494, Abcam). Membranes were washed with PBST three times for 10 min and then incubated with a secondary goat anti-rabbit or anti-mouse antibody (1:2000) for 1 h at 37°C. Subsequently, membranes were washed with PBST three times for 10 min each time. Enhanced chemiluminescence Western blotting detection regent (GE Healthcare, Chicago, IL, U.S.A.) was used to develop the blots. The intensity of the detected protein was quantified by Image Lab Software (version 4.1; Bio-Rad Laboratories, Inc.).

### Statistical analysis

Data are presented as the mean ±  standard deviation. One-way analysis of variance (ANOVA) following Turkey’s text was used to compare the variables among the different treatment groups. Statistical analysis was performed using SPSS 18.0 software (SPSS, Inc., Chicago, IL, U.S.A.), and *P*<0.05 was considered to indicate a statistically significant difference.

## Results

### Characterization of drug-loaded microbubbles

**Figure 1:**
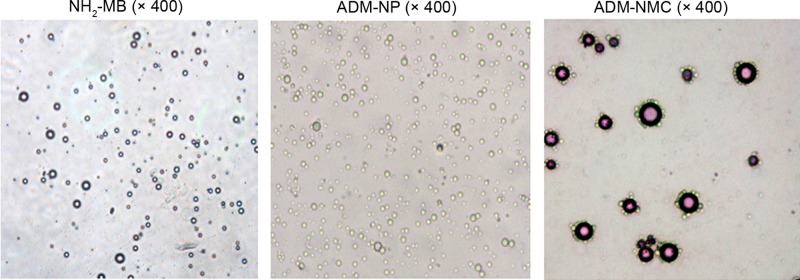
NH_2_-MB, ADM-PLGA and ADM-NMC, as visualized under a light microscope (magnification, ×400) ADM-NMC; adriamycin-loaded PLGA nanoparticle microbubble complexes; ADM-PLGA, adriamycin-loaded polylactic-co-glycolic acid nanoparticles; NH_2_-MB, lipid microbubbles.

**Figure 2:**
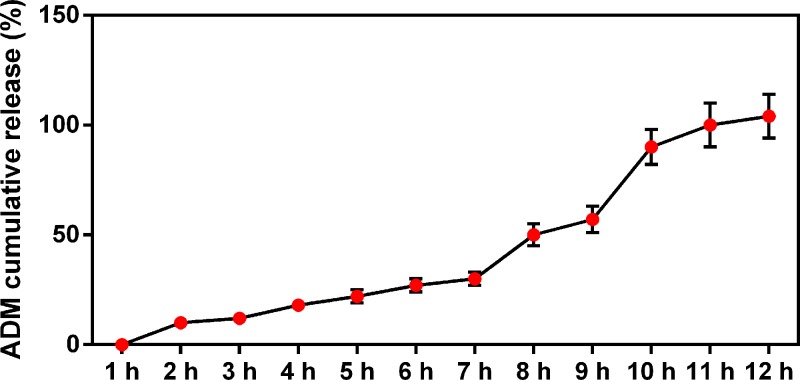
The graph of the accumulative release rate of ADM by microbubbles The release rate of ADM increased over time. The drug-loading rate of this ADM sustained-release microsphere was 7.91 ± 0.27%; ADM, adriamycin.

ADM-NMC were synthesized by conjugating NH_2_-MB with ADM-PLGA. NH_2_-MB, ADM-PLGA and ADM-NMC were visualized using a light microscope. NH_2_-MB was spherical, with a uniform size, smooth surface and uniform dispersion (Figure 1). ADM-PLGA were homogeneous in size and equally distributed (Figure 1). ADM-NMC exhibited a large number of ADM-PLGA gathered around NH_2_-MB in garland-like manner (Figure 1). The release of ADM from ADM-NMC was increased with time increasing (Figure 2).

### Effect of drug-loaded microbubbles on the effects of ultrasound image rabbit hepatic VX2 tumor and tumor volume

**Figure 3:**
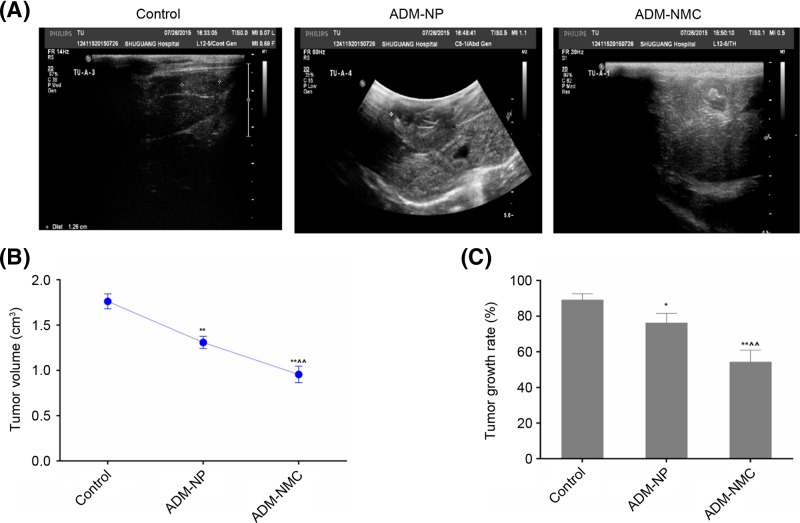
Effect of drug-loaded microbubbles on the effects of ultrasound image rabbit hepatic VX2 tumor and tumor volume (**A**) Ultrasound images of the control, ADM-NP and ADM-NMC groups. (**B**) Tumor volume in the control, ADM-NP and ADM-NMC groups, which were 1.77 ± 0.071 cm^3^, 1.36 ± 0.063 cm^3^ and 0.92 ± 0.021 cm^3^, respectively. (**C**) Tumor growth rate of the control, ADM-NP and ADM-NMC, groups, which were 89.32 ± 3.44%, 76.32 ± 5.34% and 54.42 ± 6.48%. Data are presented as the mean ± standard deviation; *n*=5. **P<0.01 vs. control group; ^∧∧^*P*<0.01 vs. ADM-NP group. ADM-NMC, adriamycin-loaded PLGA nanoparticle microbubble complex group; ADM-NP group, adriamycin-loaded PLGA nanoparticle group; PLGA, polylactic-co-glycolic acid.

The ADM-NP and ADM-NMC diluted with physiological saline were injected into rabbits in the ADM-NP and ADM-NMC groups, respectively, via the ear vein. The control group was injected with saline alone. Images of the rabbit hepatic VX2 tumor in three groups were obtained using ultrasound (Figure 3A). The echogenicity was obvious in the interior part of the tumors. Ultrasound scanning indicated that the tumor volume of all three groups increased to some degree following treatment. Tumor volume in the control, ADM-NP and ADM-NMC groups were 1.77 ± 0.071 cm^3^, 1.36 ± 0.063 cm^3^ and 0.92 ± 0.021 cm^3^ respectively (Figure 3B). Tumor volume in the ADM-NMC group was significantly lower than in the control and ADM-NP groups (*P*<0.01; Figure 3B).

### Effect of drug-loaded microbubbles combined with ultrasound on the tumor

**Figure 4:**
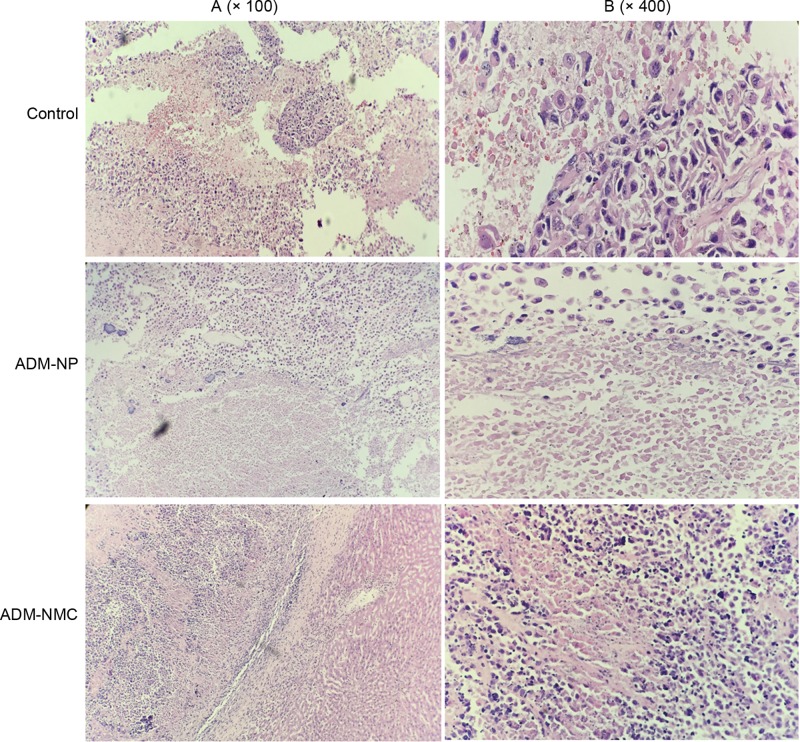
Light microscopy images of tumor sections stained with hemotoxylin and eosin (**A**) Light microscopy images of the control, ADM-NP and ADM-NMC groups (magnification, ×100); scale bar, 50 μm. (**B**) Light microscopy images of the groups (magnification, ×400); scale bar, 15 μm; ADM-NMC, adriamycin-loaded PLGA nanoparticle microbubble complex group; ADM-NP group, adriamycin-loaded PLGA nanoparticle group; PLGA, polylactic-co-glycolic acid.

Tumor growth rate was calculated from the ultrasonic imaging. The tumor growth rate was 89.32 ± 3.44%, 76.32 ± 5.34% and 54.42 ± 6.48% in the control, ADM-NP and ADM-NMC groups, respectively (Figure 3C). The tumor growth rate in the ADM-NC group was significantly lower than in the ADM-NP and control groups (*P*<0.01; Figure 3C). Furthermore, the tumor shape in control group showed tumor cell of the aggregation to form a solid nest-like or alveolar structure. Besides, the pathologic structure of tumor tissues in ADM-MNC group and ADM-NP group becomes denser (Figure 4).

### Effect of drug-loaded microbubbles combined with ultrasound targeted microbubble destruction on apoptosis

**Figure 5:**
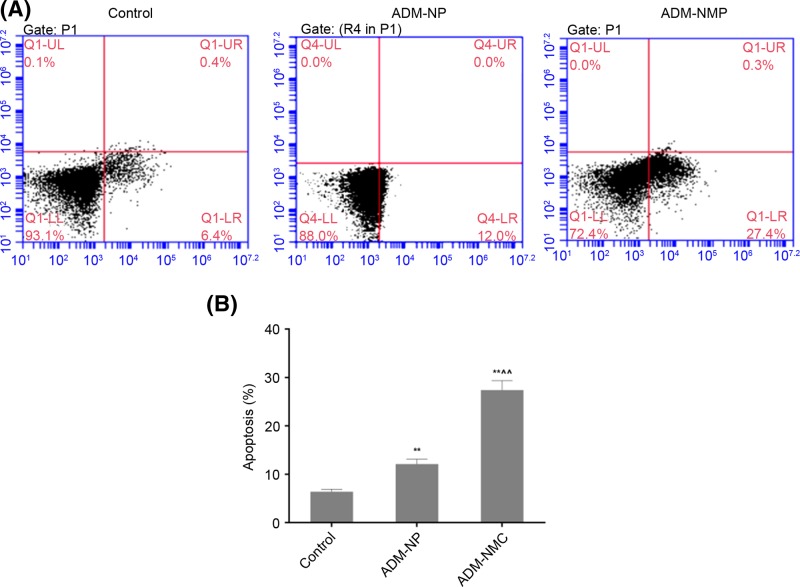
Effect of drug-loaded microbubbles combined with ultrasound on tumor cell apoptosis (**A**) Evaluation of tumor cell apoptosis by flow cytometry. (**B**) Quantification of the apoptosis rate. Data are presented as the mean ± standard deviation; *n*=5, **P<0.01 vs. control group; ^∧∧^*P*<0.01 vs. ADM-NP group. ADM-NP group, adriamycin-loaded PLGA nanoparticle group; ADM-NMC, adriamycin-loaded PLGA nanoparticle microbubble complex group; PLGA, polylactic-co-glycolic acid.

**Figure 6:**
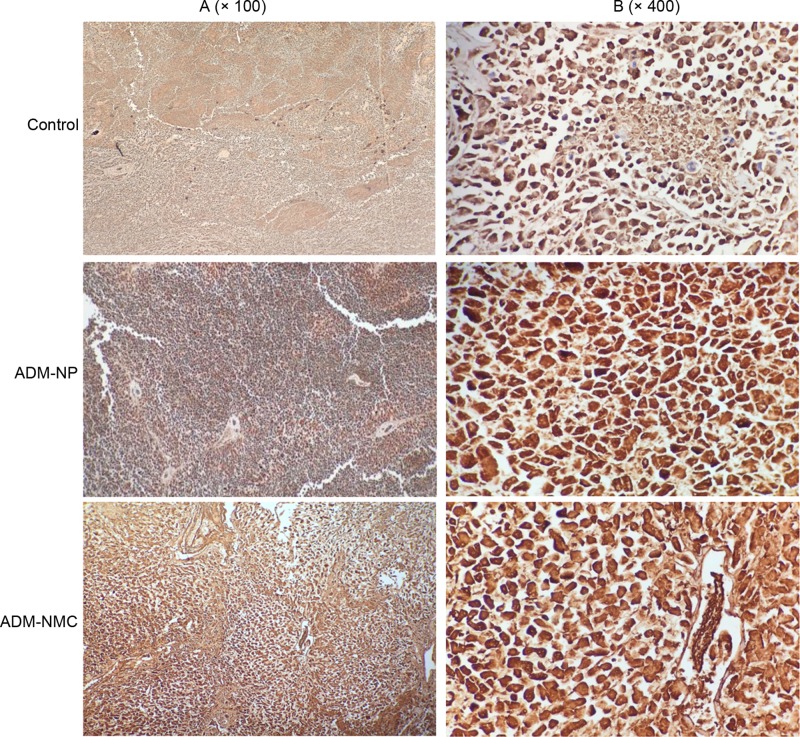
Immunohistochemical staining of tumor sections for Bax Positive staining of Bax was indicated by granular brown staining primarily located in the cytoplasm. (**A**) Light microscope images of the control, ADM-NP and ADM-NMC groups (magnification, ×100); scale bar, 100 μm. (**B**) Light microscope images of the control, ADM-NP and ADM-NMC groups (magnification, ×400); scale bar, 15 μm. ADM-NP group, adriamycin-loaded PLGA nanoparticle group; ADM-NMC, adriamycin-loaded PLGA nanoparticle microbubble complex group; Bax, B cell lymphoma-2-associated X protein; PLGA, polylactic-co-glycolic acid.

**Figure 7:**
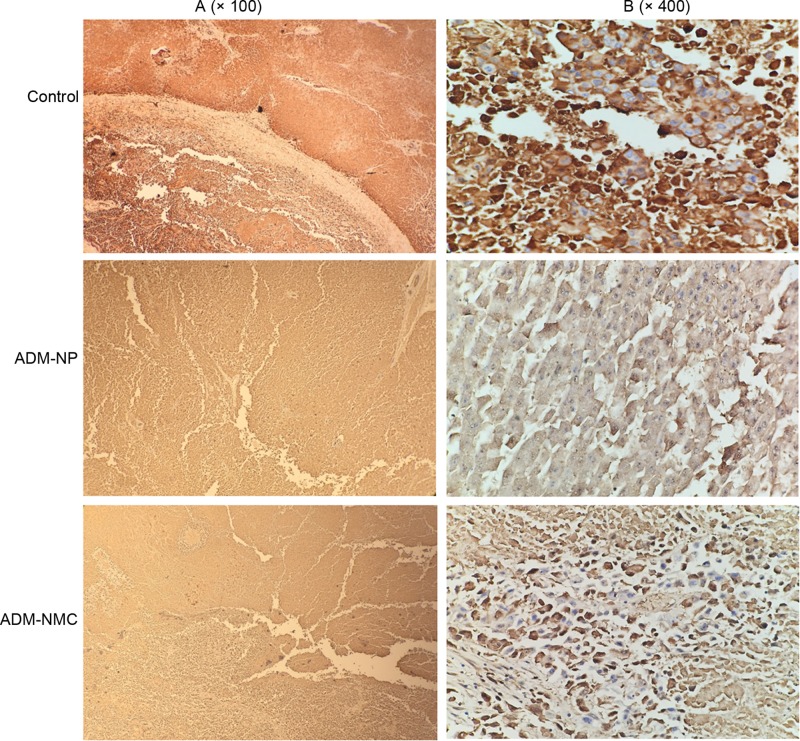
Immunohistochemical staining of tumor sections for Bcl-2 Positive expression of Bcl-2 was indicated by brown intracytoplasmic granules. (**A**) Light microscope images of the control, ADM-NP and ADM-NMC groups (magnification, ×100); scale bar, 100 μm. (**B**) Light microscope images of the control, ADM-NP and ADM-NMC groups (magnification, ×400); scale bar, 15 μm. ADM-NMC, adriamycin-loaded PLGA nanoparticle microbubble complex group; ADM-NP group, adriamycin-loaded PLGA nanoparticle group; Bcl-2, B-cell lymphoma-2; PLGA, polylactic-co-glycolic acid.

**Figure 8:**
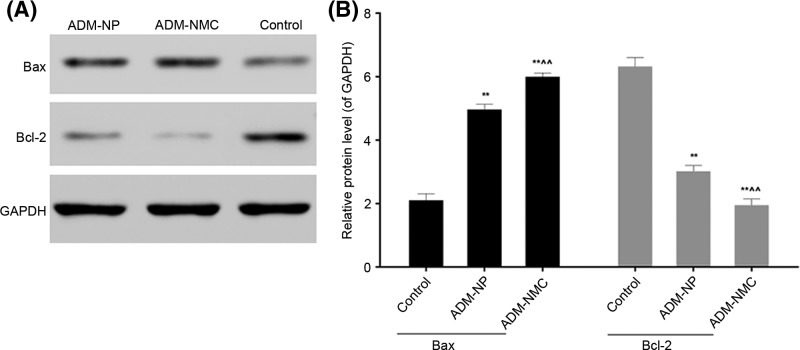
Effect of drug-loaded microbubbles combined with ultrasound targeted destruction on the expression of Bax and Bcl-2 (**A** and **B**) Bax and Bcl-2 were detected by Western blotting. The expression of Bax was increased and expression of Bcl-2 was decreased following treatment with ADM-NP and ADM-NMC. GAPDH was used as a loading control. Data are presented as the mean ± standard deviation, *n* = 5, **P<0.01 vs. control; ^∧∧^*P*<0.01 vs. ADM-NP group. ADM-NP group, adriamycin-loaded PLGA nanoparticle group; ADM-NMC, adriamycin-loaded PLGA nanoparticle microbubble complex group; Bax, Bcl-2-associated X protein; Bcl-2, B-cell lymphoma-2; PLGA, polylactic-co-glycolic acid.

Liver cancer cell apoptosis was analyzed using flow cytometry (Figure 5), which indicated that the apoptosis rates in the ADM-NP and ADM-MNC groups were significantly higher than in the control group (*P*<0.01). Furthermore, the apoptosis rate of the ADM-MNC group was significantly greater than that of the ADM-NP group (*P*<0.01; Figure 5B). The apoptosis rates were 6.4 ± 0.52%, 12.1 ± 1.02% and 27.4 ± 1.94% in the control, ADM-NP and ADM-MNC groups, respectively. Bax and Bcl-2 are proteins associated with apoptosis. Positive expression of Bax (Figure 6) and Bcl-2 (Figure 7) was identified in the cytoplasm, indicated by the presence of brown intracytoplasmic granules. The expression of Bax in the ADM-NP and ADM-NMC groups was significantly increased compared with the control group (*P*<0.01; Figure 8). By contrast, the expression of Bcl-2 protein was significantly decreased in the ADM-NP and ADM-NMC groups compared with the control group (*P*<0.01; Figure 8). Furthermore, in the ADM-NMC group, the expression of Bax protein was significantly higher and the expression of Bcl-2 protein was significantly lower than in the ADM-NP group (*P*<0.01; Figure 8).

## Discussion

Drug-loaded microbubbles combined with ultrasound facilitated the release of drugs into the bloodstream that targeted a specific area and remained in the bloodstream for a more prolonged period compared with drugs administered using conventional methods [15]. Lipid microbubbles acting as drug carriers were released in a particular location under the guidance of ultrasound. These drug-loaded microbubbles were burst in the target region and the drugs were locally released using ultrasound irradiation. Using such an approach may promote the permeability of drugs by enhancing the permeability of local microvessels and cell membranes [16]. Following the release of the drugs close to the lesion, the drug concentration increased temporarily and was readily affected by various factors, including temperature and fluid shear stress. These were limiting factors in its clinical application [17,18].

PLGA possess numerous advantages compared with other drug carriers. It has a small volume, effectively releases drugs in a sustained manner, remains in the circulation for a long time and exhibits passive targeting ability. These characteristics may decrease the quantity of chemotherapeutic drugs required and decrease the toxic side effects associated with their use [18]. The efficiency of PLGA was restricted by multiple physical and chemical factors [19]. The *in vivo* environment was complicated to work in and it was difficult to assemble high concentrations of the drug carrier [17]. However, ADM-NMC and ADM-PGLA were successfully synthesized using lipid microbubbles and lipid microbubbles and PLGA, respectively (Figures 1 and 2).

HCC is one of the most common types of cancer and has a poor prognosis and high recurrence rate [1]. It has been demonstrated that the rabbit model with VX2 liver tumors is suitable for performing an interventional experimental study [20]. The rabbit VX2 carcinoma model was successfully constructed using the tumor block transplanting method and this was confirmed by contrast-enhanced ultrasound (Figure 3A). This was a suitable model used to test the effects of ADM-NMC on rabbits exhibiting liver tumors. The results demonstrated that the growth of tumors was suppressed in the ADM-NP and ADM-NMC groups compared with the control (Figure 3B and C). Furthermore, the tumor shape in the ADM-NP and ADM-NMC groups became more normal following treatment (Figure 4). The results of the present study demonstrated that the therapeutic effects of ADM-NMC were more pronounced than those of ADM-NP. The antitumor effect of ADM-NMC may be due to the unique characteristics and sound cavitation of ultrasound and the enhanced permeability and retention effect on tumor tissue [21,22]. In ADM-NMC, ADM-NP was closely linked to NH_2_-MB via covalent binding, microbubble fragmentation exerted a cavitation effect under ultrasonic irradiation and more nanoparticles were released into the target tissue. This demonstrates that the use of drug-loaded microbubbles combined with ultrasound may achieve optimal therapeutic results. However, its method of action remains unclear and further studies are required.

The induction of apoptosis is one of the most basic approaches of treating cancer and liver cancer is no exception. In the present study, the morphology of tumor tissue improved as the apoptosis rate of liver cancer cells increased following treatment with ADM-NP and ADM-NMC (Figures 4 and 5). It was suggested that ADM-NMC helped induce liver cancer cell apoptosis. Bax is a pro-apoptotic protein, whereas Bcl-2 is an anti-apoptotic protein [13,14] and immunostaining for these proteins indicate that they were present in the cytoplasm of tumor cells (Figures 6 and 7). The expression of Bax protein was significantly up-regulated and the expression of Bcl-2 protein was significantly down-regulated by ADM-NMC compared with the ADM-NP and control groups (*P*<0.01; Figure 8). This demonstrates that ADM-NMC regulates the expression of Bax and Bcl-2. The initiation and progression of carcinoma may be viewed as the destruction of the homeostasis between tumor cell proliferation and apoptosis [23]. When tumor cell apoptosis is higher than tumor cell proliferation, tumor growth is suppressed. The results of the present study indicate that ADM-NP and ADM-NMC may induce apoptosis in liver cancer cells and that ADM-NP has a greater effect than ADM-NC. This suggests that liver cancer cell apoptosis may be induced by ADM-NMC via regulation of Bax and Bcl-2 expression.

In conclusion, the present study determined that the use of drug-loaded microbubbles combined with ultrasound may effectively inhibit tumor growth by promoting the apoptosis of cancer cells via regulation of Bax and Bcl-2 expression.

## Availability of data and materials

All data generated and/or analyzed during this study are included in this published article.

## Abbreviations

ADM: adriamycin
ADM-NMC: adriamycin-loaded PLGA nanoparticles microbubble complexes
ADM-NP: adriamycin-loaded PLGA nanoparticles
Bax: Bcl-2-associated X protein
Bcl-2: B-cell lymphoma-2
DPPC: dipalmitoyl phosphatidylcholine
HCC: hepatocellular carcinoma
PLGA: polylactic-co-glycolic acid
